# Performance of focus-tunable presbyopia correction lenses operated using gaze-tracking and LIDAR

**DOI:** 10.1364/BOE.543807

**Published:** 2025-02-03

**Authors:** Rajat Agarwala, Björn R. Severitt, Felix F. Reichel, Benedikt W. Hosp, Siegfried Wahl

**Affiliations:** 1 ZEISS Vision Science Lab, Institute for Ophthalmic Research, University of Tübingen, Maria-von-Lindenstr. 6, Tübingen, Germany; 2 University Eye Hospital, Centre for Ophthalmology, University Hospital Tübingen, Tübingen, Germany; 3 Carl Zeiss Vision International GmbH, Turnstr. 27, Aalen, Germany

## Abstract

Presbyopia is an age-related loss of accommodation ability of the eye which affects an individual’s capacity to focus on closer objects. With the advent of tunable lens technologies, various algorithms have been developed to tune such lenses for presbyopia correction in older populations. In this study, we assessed a gaze and LIDAR-based feedback mechanism with electronically tunable lenses for their use as correction lenses for presbyopia. The tunable lens prototype was evaluated in 15 healthy young participants with their corrected sphero-cylindrical refraction by comparing their performance for a dynamic matching task under two conditions: (1) natural accommodation, and (2) emulating presbyopia using cycloplegic drops to paralyse accommodation while focussing using the developed visual demonstrator prototype. The participants performed the matching task on three screens placed at multiple distances. We have demonstrated that gaze can be used in conjunction with LIDAR to tune the lenses in the wearable visual demonstrator prototype, enabling participants to achieve a fast and accurate response for the matching task.

## 1. Introduction

Presbyopia, resulting from the stiffening of the eye lens or extra-lenticular changes, incapacitates the ability of the eye to focus on near objects and concerns every human observer [1]. Presbyopia is typically diagnosed in individuals during their forties or early fifties, when they first experience difficulty with near vision tasks such as reading small print or viewing their phones at a close range. The current methods of presbyopia correction include various static forms of spectacles such as reading glasses and progressive addition lenses, or contact lens options such as monovision or simultaneous-vision, or intraocular lens [2,3]. However, one of the most commonly used presbyopia correction is progressive lens in a spectacle frame [4]. Presbyopes that use progressive lenses correction are unsatisfied due to the veering from natural accommodation as well as reduction in edge contrast sensitivity and ability to differentiate between depths [5]. A continuous change in dioptric power along a predefined path along a static lens inevitably leads to distortions and astigmatic blur in the periphery of the lenses [6]. Furthermore, natural gaze behaviour is strongly impacted, as the head position is determined by the intended viewing distance [7]. Many wearers experience the "swim effect," causing unnatural motion perception [8] and sometimes nausea [9]. Due to these issues, many users reject current forms of presbyopia correction [10].

Recently, researchers have shown early presbyopia correction methods using switchable or tunable lenses with liquid crystals or liquid membrane technology to provide additional dioptric power for near vision. Hasan et al. (2017) implemented varifocal liquid lens design using a piezoelectric actuation mechanism to change the optical power of the lens [11]. An adaptive eyeglass design was also implemented using this liquid lens in combination with a time-of-flight (TOF) distance sensor [12], which relied on the person to move the head to focus on objects. Liquid membrane-based lenses have been used to demonstrate tuning of focus for correcting presbyopia by Mompeán et al. (2020) and Padmanaban et al. (2019) with different sizes of clear aperture [13,14]. In the work by Mompeán et al. (2019), convergence of the participant’s eyes was used to tune the lenses after calibration and the pupil tracking was performed on a smartphone [13].The study by Padmanaban et al. (2019) used a fusion algorithm which shows good performance; However, there is still room for improvement in achieving responses that closely mimic natural accommodation, and the stereo camera’s distance range has some limitations [14]. In our previous study, a liquid membrane lens with 30 mm clear aperture was evaluated using a Hartmann-Shack wavefront sensor, and visual acuity and contrast sensitivity were measured in participants, along with feasibility being shown for use of a LIDAR camera as a control for a presbyopia correction visual demonstrator [15].

Newer tunable lens technologies have been developed and tested using cameras on optical benches to test image quality for various tuning mechanisms, such as electro-optic diffractive multifocal lenses have been demonstrated [16]. Similarly, Bhowmick et al. (2023) have used a combination of three liquid crystal lenses which enable changing of defocus and astigmatism [17].

In this study, an adaptive control algorithm for driving the tunable lenses was implemented using a solid-state LIDAR (Light Detection and Ranging) camera in tandem with gaze tracking to determine the point of focus of the user and estimate the required focus distance. To evaluate the performance of the visual demonstrator prototype with the aforementioned method, the response time and accuracy of young healthy participants with natural accommodation was compared to using the visual demonstrator while being cyclopleged using a dynamic matching task.

## 2. Materials and methods

### 2.1. Visual demonstrator prototype

**Fig. 1.**
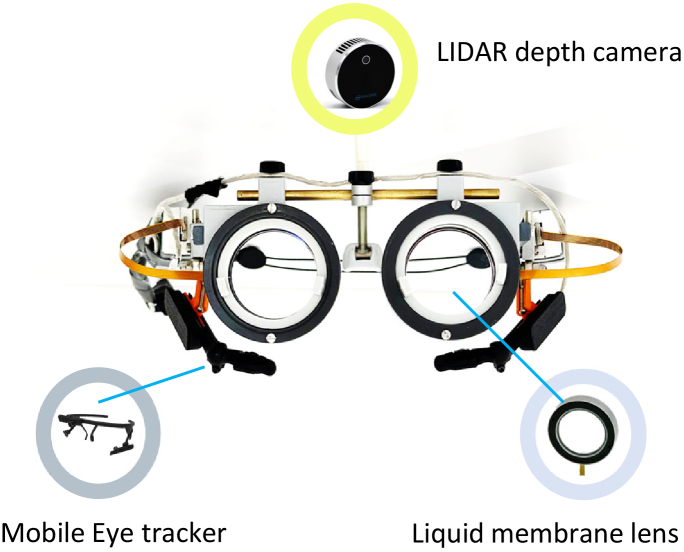
Visual demonstrator prototype equipped with eye-tracker, LIDAR camera and tunable lenses.

A spectacle frame, designed in-house, similar to a standard optical trial test frame design, fitted with a new gravity-compensated generation of tunable lenses (EL-35-45, Optotune AG, Switzerland), eye-tracker cameras (Pupil Core, Pupil Labs, Berlin, Germany) and a LIDAR camera (L515, Intel Corporation, Seattle, USA) constitute the visual demonstrator for this study (Fig. 1). The visual demonstrator has been designed keeping in mind various head sizes and facial geometry, and straps have been provided for securing it and making it comfortable for the participant. Furthermore, it preserves the features of our previous generation visual demonstrator [15] to enable adjustment of inter-pupillary distance (IPD) and lens height.

The integration of the eye tracker was done by designing custom holders for the eye tracker cameras in the frame. For the world camera of the eye tracker, the RGB (red-green-blue) camera from the LIDAR camera module was used. This approach reduced complexity as the depth sensor and the RGB camera were located in a single device and enabled simplified temporal and spatial synchronization between the two input streams of the depth and world camera. The LIDAR camera has a depth sensor for distance measurement from any point in its field of view, and it was placed on the wearer’s forehead. The LIDAR camera enabled the generation of depth maps over a field of view of 70° x 55°  with 1920 x 1080 resolution, providing information about the distance of each point from the wearer of the prototype. Both the LIDAR depth sensor and the RGB camera has a sampling rate of 30 frames per second.

### 2.2. Test setup and tuning algorithm

**Fig. 2.**
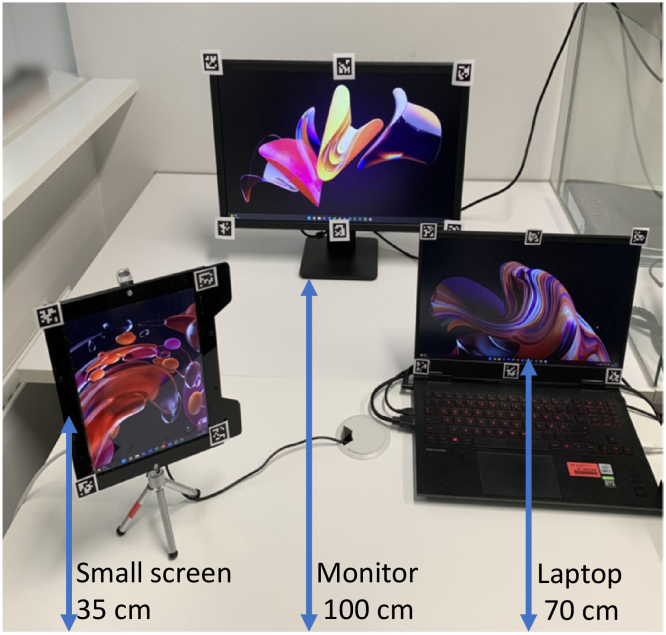
Experimental setup with three screens placed at three different distances (35 cm, 70 cm and 100 cm) from the participant.

A test setup with three screens placed on a tabletop at different distances (35, 70 and 100 cm) was used to validate the algorithm with human participants (Fig. 2) wearing the visual demonstrator prototype. Each screen had printed fiducial markers [18] placed on them for their detection in the scene. The three surfaces were defined by tagging the respective fiducial markers using the Pupil Labs Capture software. An initial eye tracker calibration was performed for accuracy and precision measurements of the eye tracker for each participant. A script was implemented in Python using open-source software PsychoPy [19] to handle stimulus display for different tasks and "Pupil-Core Network-API" to enable synchronization using system timestamps (64-bit floating-point values) of input data streams from the two eye tracker cameras, RGB camera of LIDAR module and depth stream of the LIDAR module. The distance of the wearer from the coordinates of the current gaze position on a surface was retrieved from LIDAR point-cloud data, and its reciprocal was used for tuning the dioptric power of the tunable lenses.

## 3. Assessment of the feedback system in human participants

### 3.1. Participants and pre-measurements

To assess the performance of the newly developed feedback system for controlling the tuning of the lenses in the prototype, fifteen healthy individuals participated from the University of Tübingen. The participants had an average age of 25.07± 3.94 years, and their ages ranged from 20 to 35 years. Participants with ocular pathologies, corneal laser surgery or other ocular health issues were excluded. The experimental procedures adhered to the principles outlined in the Declaration of Helsinki of 1964, and approval for this investigation was obtained from the ethical board committee of the University of Tübingen. Prior to their participation, all participants provided informed consent after a detailed explanation of the pharmaceutical agents and measurements, including indications and potential consequences. After signed consent forms were obtained, the following device-related and eye doctor examinations were performed as preliminary tests to rule out any exclusion criteria and ensure safety of the participants:

• Objective measurement of the refractive error of the eye using wavefront aberrometry (ZEISS iProfiler Plus, Carl Zeiss Vision International GmbH, Aalen, Germany).
• Sphero-cylindrical refractive correction of the currently used spectacle lenses (if any) by the use of a digital lensmeter (ZEISS Visulens 500, Carl Zeiss Vision International GmbH, Aalen, Germany).
• Measurement of the inter-pupillary distance (IPD) using a Pupilometer.
• Measurement of eye pressure and anterior chamber angle to avoid any contraindications towards the cycloplegic agent (Cyclopentolate, Alcon Ophthalmika GmbH, Stella-Klein-Lüw-Weg 17, 1020 Vienna) were performed by the eye care professional (FFR). Furthermore, an initial anamnesis ensured that the participants fit the inclusion criteria.

For each participant, the prototype was individualized by adjusting the frame to their individual inter-pupillary distance (IPD) and sphero-cylindrical refractive correction using trial lenses (Trial Lens Cases BK 1; Oculus GmbH, Wetzlar, Germany), ensuring these adjustments were maintained for the entire course of the matching task. To emulate a presbyopic condition in young participants, accommodative ability was inhibited through the administration of a cycloplegic agent. Prior to the participant commencing the matching task under the visual demonstrator condition, the eye care professional (FFR) administered the cycloplegic agent. Also, for each participant, it was ensured with the help of a push-up test that the participant had paralysed accommodation.

### 3.2. Subjective evaluation of the tunable lenses

The participants performed visual acuity and contrast sensitivity measurements while wearing the prototype without the new control algorithm, initially to test the subjective performance of the larger tunable lenses. Participants were instructed to perform the test using a standard tool, the Freiburg Visual Acuity and Contrast Test (FrACT - version 3.10.5). The optotypes were shown on an external display (Retina Display, Apple Inc., California, USA) controlled using a personal computer (OMEN Laptop 15-ek0xxx, HP Deutschland GmbH, Germany). Landolt-C rings in eight orientations with varying size were used as optotype and the participant was asked to provide feedback using a keyboard for choosing the correct orientation. The measurements were performed at three different distances (1.00 D, 1.25 D and 2.50 D) by moving the screen to that distance. The focus of the tunable lens was set to the testing distance. Similarly, the contrast sensitivity test was performed with the screen placed at 1 meter from the participant wearing the prototype. In this test, the size of the optotype remained constant however, the contrast varied based on the participant’s response. The focus was fixed to the test distance of 1 meter during this measurement.

### 3.3. Matching task

**Fig. 3.**
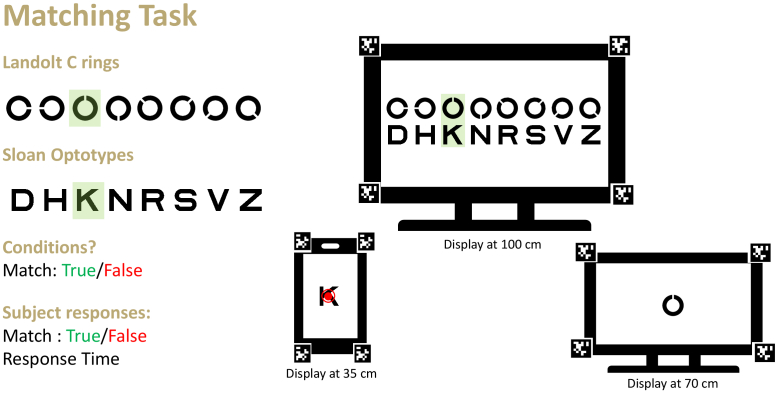
Experimental paradigm with three screens placed at different distances from the participant, depicting an example trial for a true match condition. The randomly chosen optotypes have been highlighted in green. For each trial, the screen on which a particular type of optotype and matching table appears, is randomized.

We developed a matching task based on the task developed by Hosp et al. [20] for our current test setup to evaluate the functioning of the visual demonstrator prototype (Fig. 3). A set of 8 Landolt-C rings (opening in one of the eight directions) and 8 Sloan letters (D, H, K, N, R, S, V, Z) were chosen as optotypes for this task. For each trial, one of the eight randomly chosen Landolt-C rings was shown on the first screen and a randomly chosen Sloan letter was shown on the second screen. On the third screen, a matching table with all the optotypes were shown. It was arranged in two rows, with Landolt-C rings in the first row and Sloan optotypes in the second row. The elements were shuffled in the matching table for each trial to avoid memorization of the combinations of the optotypes by the participants. The screen on which the type of stimulus would appear, such as Landolt-C rings, Sloan letters and the matching table, was randomized for each trial. If the optotypes displayed on the first or second screen should be shown as a match condition in the matching table, i.e. present in a single column, had a 50% chance of occurrence. The participants had to answer if the individual stimuli on two screens were present in a single column in the third screen or not, thereby identifying a match or non-match, by responding using the keyboard by ’y’ or ’n’.

The participants performed the matching task under two conditions: initially, with their natural accommodative ability intact and wearing their sphero-cylindrical refractive correction; and subsequently, following cycloplegia, using a visual demonstrator to focus at varying distances and wearing their sphero-cylindrical refractive correction. Participants were presented with stimuli on the three screens at three different distances in their field of view, requiring them to change their gaze dynamically. For the latter condition, they could only see the stimuli if the power of the tunable lenses was tuned to the right distance due to their paralysed accommodative ability. Manual adjustments for detection parameters for minimum and maximum radii of pupils were performed in the Pupil core software to enable better detection of larger pupils due to cycloplegia. The range of the pupil radii was adjusted until the blue circle for the eye model detection in pupil labs software appeared. A dark blue circle represents that the eye model fitted to the participant’s eye is within the physiological bounds and can be used for gaze tracking. The participants performed 3 repetitions for each condition. For each repetition, 30 trials were performed by the participants. In total, the participants performed 180 trials, i.e. 90 trials for each condition. For all the trials, the accuracy and timing data for the matching task were recorded, along with the simultaneous collection of the eye-tracking, tunable lens power, and LIDAR data.

### 3.4. Usability test

After performing the matching task with the visual demonstrator prototype, the participants undertook a User Experience Questionnaire (UEQ-S) designed to assess task-related (pragmatic) and non-task-related (hedonic) aspects [21]. The participants were provided a table (Table. 1) containing 8 parameters that they needed to rate on a scale with 7 levels. The participants were instructed to fill this test based on their experience with tuning of the visual demonstrator prototype during the matching task. During the analysis, values are converted to a range from -3 to +3, with +3 indicating the most positive response and -3 indicating the most negative response.

**Table 1.** Usability Evaluation Questionnaire for evaluating user experience with the visual demonstrator prototype.

Negative Attribute	Scale	Positive Attribute	Category

Obstructive	( ) ( ) ( ) ( ) ( ) ( ) ( )	Supportive	Pragmatic
Complicated	( ) ( ) ( ) ( ) ( ) ( ) ( )	Easy	
Inefficient	( ) ( ) ( ) ( ) ( ) ( ) ( )	Efficient	
Confusing	( ) ( ) ( ) ( ) ( ) ( ) ( )	Clear	

Boring	( ) ( ) ( ) ( ) ( ) ( ) ( )	Exciting	Hedonic
Not interesting	( ) ( ) ( ) ( ) ( ) ( ) ( )	Interesting	
Conventional	( ) ( ) ( ) ( ) ( ) ( ) ( )	Inventive	
Usual	( ) ( ) ( ) ( ) ( ) ( ) ( )	Leading edge	

## 4. Results

### 4.1. Optometric measurements

The results of the optometric measurements, including spherical refraction data, eye pressure, pupillary distances, visual acuity and contrast sensitivity are summarized below (Table 2).

**Table 2.** Summary of optometric measurements

Measurement	Right eye (OD)	Left eye (OS)	Binocular

Objective Refraction (D)	−1.03±1.26	−0.92±1.20	-
Sphero-cylindrical Refraction (D)	−0.98±1.31	−0.87±1.18	-
Far Pupillary distance (mm)	31.33±1.35	31.47±1.27	62.8±2.27
Near Pupillary distance (mm)	28.23±1.03	28.27±1.18	56.5±1.63
Eye Pressure (mmHg)	11.67±1.88	11.93±2.55	-
Mean Visual Acuity (logMAR)	−0.19±0.10	−0.19±0.06	−0.28±0.03
Mean Contrast Sensitivity (logCS)	1.92±0.11	1.94±0.07	2.11±0.06

### 4.2. Matching task performance

**Fig. 4.**
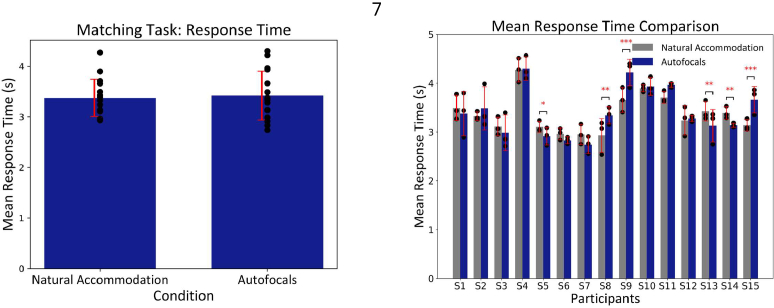
Mean response time comparison for the matching task between natural accommodation and autofocal conditions. (A) The bars represent the mean response time across all participants, and the red error bar represents the standard deviation. Each point represents the mean response time over the 90 trials, i.e., three repetitions for every individual participant. (B) The bars represent the mean response time for each individual participant for each condition, and the red error bar represents the standard deviation. Each point represents the mean response time over 30 trials, i.e., one repetition for each participant. Asterisks indicate statistical significance: *:p < 0.05, **:p < 0.01, ***:p < 0.001.

For the response time comparison, the participants performed the matching task with a mean of 3.37±0.37 seconds and 3.42±0.48 seconds for natural accommodation and autofocal conditions, respectively (Fig. 4(A)). A paired t-test was performed, and no significant differences were observed in participants’ response time between the two conditions (t= −0.63 , p= 0.53 (two-tailed)). In case of individual participants, it was observed that a few participants (S4, S9, S10, S11) needed longer than the mean response time to perform the matching task, but their individual performance was similar between the two conditions (Fig. 4(B)). Furthermore, in case of T-tests performed for individual participants, it was observed that there were significant differences between the two conditions for participants S5, S8, S9, S13, S14 and S15 (Fig. 4(B)).

**Fig. 5.**
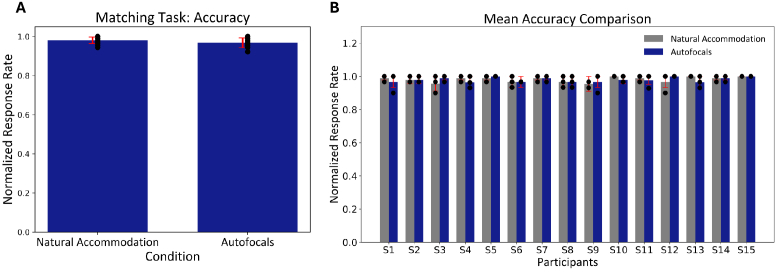
Mean accuracy comparison for the matching task between natural accommodation and autofocal conditions. (A) The bars represent the mean accuracy across all participants and the red error bar represents the standard deviation. Each point represents the mean accuracy over the 90 trials, i.e., three repetitions for every individual participant. (B) The bars represent the mean accuracy for each individual participant for each condition, and the red error bar represents the standard deviation. Each point represents the mean accuracy over 30 trials, i.e., one repetition for each participant.

The mean accuracy was similar between the two conditions with 0.980±0.016 and 0.968±0.024 number of normalized counts over repetitions for natural accommodation and autofocal conditions, respectively (Fig. 5). Similar to response time, there was no significant difference observed between the two conditions for a paired t-test (t= 1.51 , p= 0.15 (two-tailed)).Furthermore, the participants demonstrated that they performed the matching task with no significant difference in terms of accuracy between the two conditions individually (Fig. 5).

### 4.3. Usability test

**Fig. 6.**
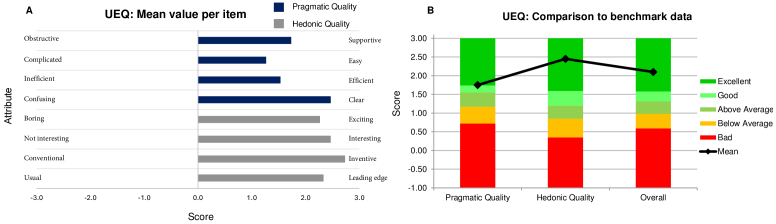
(A) Results of the User Experience Questionnaire from the present study and (B) its comparison to existing benchmark data in terms of pragmatic and hedonic characteristics.

The subjective user ratings for the use of the visual demonstrator prototype to focus on various screens while having paralysed accommodation demonstrate that the participants rated the device positively for all the pragmatic and hedonic qualities (Fig. 6(A)).

The mean ratings were 1.75, 2.45 and 2.10 for the pragmatic quality, hedonic quality and overall scales, respectively (Fig. 6(B)). Raw user ratings alone provide limited insight, but comparing the results to a benchmark provides better understanding of the ratings [21]. An annually updated UEQ benchmark data from the official providers of the usability questionnaire was chosen for comparison, which is based on 452 product evaluations and data from over 20,190 participants [21]. The user ratings from this current study fall into the excellent category, placing them in the top 10 % results of the UEQ usability questionnaire.

## 5. Discussion

The field-of-view (FOV) for the lenses is an important aspect to consider for any spectacle correction to enable a wider clear view for the user. In case of previous research with 16 mm clear aperture for liquid membrane lenses, the placement of the lenses had to be in line with the centre of the pupil and then the periphery was blocked by the rim of the tunable lens [13]. The tunable lenses used in this study could be placed at a distance of about 2 to 3 cm from the eyes and enabled a corresponding FOV between 50.6°and 66°, further increasing the FOV as compared to 30 mm clear aperture lenses used in the previous studies [14,15]. In the previous generation of the liquid membrane lenses, the vertical coma component was large and either a coma corrector or an improvement in lens design was necessary as compared to gravity-compensated tunable lenses in the current generation [14,22]. However, in this generation of the lenses, EL-35-45, the coma component of the higher order aberrations are even lower than the previous generations as shown in the wavefront characterization measurements.

Over the recent years, significant advancements have been made in the development of control systems for tuning various types of lenses for presbyopia correction, progressing from user-based approaches to time-of-flight sensors and gaze or pupil tracking-based methods [12–14,22]. Depth-based methods, either as standalone solutions or combined with vergence tracking, have also been explored. Each of these prototypes has contributed valuable insights and represents important milestones in the evolution of this technology. At the same time, it is important to acknowledge that challenges persist with these methods. For example, time-of-flight sensors often require head movements to tune the lens effectively, while vergence estimation using pupil tracking demands individual calibration and can be unreliable for distances beyond arm’s length [14,23]. In this study, we present a prototype that integrates gaze tracking with depth information from a LIDAR camera, addressing some of these limitations. Our approach was evaluated using a matching task designed to simulate an office scenario where participants alternated their focus between documents on a desk (0.35 m), a computer screen (0.7 m), and a person sitting across the desk (1 m). The current evaluation paradigm allowed participants to move their heads freely, creating a setup more reflective of natural head movements in the real-world. The sphero-cylindrical corrected young participants demonstrated, on average, response speeds and accuracy under cycloplegic conditions to be comparable to their natural accommodation state, exhibiting the feasibility of this system in dynamic scenarios. There were some significant differences for a few subjects when T-tests were performed for individual participants. These differences might be attributed to factors such as participant fatigue or lapses in concentration over the course of the experiment. However, some participants demonstrated improvements in response time when using the autofocal, which may suggest a potential training effect.Importantly, the prototype successfully managed real-time depth adjustments without causing discomfort from power changes, and the usability tests highlighted its user-friendliness and convenience. By building upon the foundational work of previous prototypes, our study contributes to the ongoing technological journey, offering an approach that combines strengths from earlier methods while further improving control strategies and to better understand the usability challenges of tunable lens-based presbyopia correction methods.

The eye tracking cameras used in this study required an initial calibration, however, there are commercial eye trackers such as Pupil Neon or Tobii Pro Glasses 3 that work with little or no need for calibration. The combination of gaze tracking with depth estimation can also reduce the limitations of eye tracker calibration errors [14]. This could eliminate the need for calibration for each user of the presbyopia correction, and the LIDAR depth sensor works out-of-box and does not require any additional calibration. The depth resolution of LIDAR cameras performs clearly better than that of stereo cameras [24–26] and provides over 23 million accurate depth pixels per second over the entire range needed for the required accommodative distance measurement [24]. Furthermore, one could employ other gaze tracking attributes such as fixation or saccadic movements to control the tunable lenses.

Ageing introduces some challenges in eye tracking with older adults, but these are manageable with careful consideration [27]. Longer saccade and vergence latencies, and more frequent blinks might introduce variability in tracking data [28–30]. Future work should account for these age-related changes to perform adjustments in design of the algorithm. By accounting for these factors, eye-tracking studies could still capture valuable and reliable data, even in older populations, especially when focusing on tasks involving motion and target acquisition. Also, longer latencies could mean that the system could work slower than the current algorithm and still be able to tune without discomfort to the wearer.

Various tunable lens systems for presbyopia correction have demonstrated promising capabilities in terms of low power consumption, manageable hardware bulk, and fast response times. For example, the tunable lens modalities such as liquid crystal lenses and liquid membrane lenses (current study) operate at low voltages, below 5 Volts [31,32]. Karkhanis et al. (2022) already showed that their autofocussing eyeglasses with liquid lenses could run for up to 19 hours between charge cycles [33]. Hardware bulk of the MEMS-based LIDAR sensor has been already reduced by integrating them in phone and tablet camera systems. All these technologies are constantly evolving and continuous improvements in design and optimization are necessary to fully address the challenges of power consumption, latency and dynamic performance in everyday use.

The recruitment of healthy participants ensured that they did not have different levels of residual accommodations and could be cyclopleged to paralyse accommodation. This approach enabled an analysis of the visual demonstrator prototype for the tuning mechanism independently. During the measurements, the participants relied entirely on the tunable lens to focus on the targets at the different distances. However, the current study does not account for residual accommodation found in presbyopes. Future work for presbyopia correction with tunable lenses can focus on recruiting presbyopes into groups having different levels of residual accommodation to compare the results of the device against a baseline. Furthermore, the testing environments could be expanded to other real-world situations with various lighting conditions and tasks, such as driving and daily outdoor activities. This would enable testing of the robustness of the tuning mechanism, address the technical limitations and allow for further improvements of the presbyopia correction system. Comparing the performance of the operation of tunable lenses with natural accommodation of young participants and observing comparable results underlines the fact that gaze tracking with LIDAR is a prospective method to tune lenses for presbyopia correction.

## 6. Conclusion

The observation of low wavefront errors for both lower and higher order aberrations demonstrate the feasibility of liquid membrane lenses to be used as focus tunable lenses in presbyopia correction. Furthermore, driving these lenses using an adaptive control algorithm, employing sensor fusion between gaze tracking and depth sensor, paves the path towards operating focus tunable lenses not just for presbyopia correction, but towards other applications as well. Future work is needed in the direction of stabilisation of adaptive dioptric power changes using other eye tracking parameters, multimodal data, hardware miniaturisation and power consumption reduction for realization of true potential of this presbyopia correction method.

## Data Availability

Scripts and data underlying the results presented in this paper are not publicly available at this time, but may be obtained from the authors upon reasonable request.
